# Hospital Festivals and Meetings

**Published:** 1895-06-22

**Authors:** 


### HOSPITAL FESTIVALS AND MEETINGS.

### THE METROPOLITAN HOSPITAL.

Seldom has more enthusiasm characterised a hospital dinner than that which was evinced at the annual festival of the Metropolitan Hospital, held at the Whitehall rooms of the Hotel Metropole on Thursday in last week. The Chairman had, because of the especial way in which the hospital caters for Jews, been selected from that section of the community, and admirably did Mr. Leopold de Rothschild fulfil the duties of the position; the speeches were much above the average of post-prandial deliveries; and, what is more to the point, the silence which is golden, and which manifested itself in the subscription list, was the means of raising a far larger amount than had been raised at any previous festival. Even the music deserves special mention, for the Meistersingers' orchestra were accorded a compliment we do not remember to have met with at a hospital dinner before—they were called upon to repeat one of the items in their well-selected programme. Mr. Rothschild was supported by the veteran chairman of the hospital (Mr. Joseph Fry), the Right Hon. Lord Battersea (treasurer), the High Sheriff, the Rev. Dr. Adler, Baron de Bush, and some 130 other guests. The principal toast, "Prosperity to the Hospital," was submitted early in the evening by the Chairman, who, referring to the presence of Mr. Fry, said he was the patron saint of the hospital. He had told him (the speaker) that he was 86 years of age, but he did not believe him. (Laughter.) He had the rosy cheeks of youth; he had the plentiful hair of a man of middle age, and his vigour was equally great. It was of good omen for the cure and welfare of the patients of the hospital that the patron saint was a gentleman whose age was great but whose appearance was youthful. (Renewed laughter.) Most of those present being friends of the hospital, anything he might tell them about it would, perhaps, be better known to them than it was to himself, but their excellent secretary had given him some statistics which he felt it his duty to lay before them. The hospital was founded in 1836, and was first situated in Devonshire Square until that terrible modern plague, the railroad (laughter), caused its removal. A suitable site was found in the Kingsland Road, and in the building erected 78 beds were provided, but of that number only 66 were available through want of funds, and in consequence many most urgent cases had constantly to be refused admission. In 1894 there were 781 in-patients under treatment and no less than 78,233 out-patients. In order to find the medical comforts and necessities for this vast number of patients the annual subscriptions amounted to the paltry sum of £800, and the endowment was less than £300. Those figures spoke for themselves. The hospital was therefore bound to go round and ask for subscriptions on all sides. Alluding to the Duke of Devonshire's remark on the previous day that the time might not be far distant when it might be necessary for hospitals to appeal for State aid, Mr. Rothschild said he hoped that time would never come. (Hear, hear.) Passing on to refer to the special way in which the hospital cared for his Jewish brethren, the chairman said last year there was some difficulty as to the medical treatment of poor Jews. They appealed to that hospital and they found the committee ready to receive them. Mr. Adler would testify that everything that could be done was done to safeguard the prejudices of those poor people, even a special kitchen being provided to cook their food according to the strict law of Leviticus. (Applause.) It had been his pleasure to visit the hospital that week, and he never saw a hospital which, according to his views, was conducted on better principles. After a passing allusion to the fact that Mr. Passmore Edwards had offered to defray the cost of erecting a convalescent home for patients from this hospital, the Chairman concluded with a spirited appeal to those he was addressing to do all they could to help on the good work.

Lord BATTERSEA, in responding to the toast, expatiated on the admirable way in which the Metropolitan Hospital fulfilled its duty, and the great need there was for its existence, because of the poverty of the district in which it was placed. The Jewish element was cared for more than it had ever been cared for before, and they one and all told him that nowhere had they received greater attention and greater sympathy than in that hospital. (Applause.)

The Committee of Management were toasted, on the proposition of the HIGH SHERIFF, in one of those cultured little speeches in the making of which he is a past master.

Mr. FRY, on rising to reply, met with an extremely flattering reception. He said he felt that he was shut up. (Laughter.) After the admirable speeches of the Chairman and Lord Battersea there was nothing left for him to say. He was simply an old beggar—(renewed laughter)—who had to try and get money for the hospital. The Committee were in a painful position, and they wanted liberal men to pay off their debt and give them a fresh start. He would stick to his post as long as he could, but personal application was needed in order to get subscriptions, and at his time of life that was not a very easy thing to do. (Hear, hear.)

At the close of Mr. Fry's speech the subscription list was announced by the popular SECRETARY (Mr. C. H. Byers), the total being £3,334 10s., or £300 more than had been received at any previous festival. The announcement was received with loud cheers, which, having subsided, the remaining toasts were proceeded with.

"The Hospital Staff" was submitted by Mr. C. J. THOMAS, and acknowledged by Dr. GOODSALL and Dr. TOOTH, the latter pleading for the substitution of wood pavement for the stone setts with which the road in front of the hospital is now laid.

Replying to the toast of his own health (proposed by Mr. L. DAVIDSON), the CHAIRMAN urged his co-religionists to strain every nerve to show they were deserving of the good feeling shown to them by Christians.

The last toast was that of "The Visitors," to which Dr. ADLER replied. Alluding to the appointment of Dr. Abraham Cohen as a medical officer for Jewish out-patients, the Chief Rabbi said it was not because they could not trust their brethren to Christian doctors, but because they wanted a doctor who understood their peculiarities, and who could speak Yiddish a little better than Lord Battersea or even Mr. Zangwill. (Laughter.)

### A HOSPITAL FOR BATTERSEA.

### A LIVELY MEETING.

The promoters of the meeting held on Saturday at Battersea Town Hall for the purpose of facilitating the conversion of Bolingbroke House, Wandsworth Common, into a hospital for the district could scarcely have expected a larger gathering than that which assembled, for people are loth to give up the Saturday half-holiday when fields are bathed in sunshine for a hospital meeting, but they might reasonably have anticipated greater harmony. The platform and about half the area of the fine hall were occupied when Lord Spencer rose to introduce the object of the meeting. His Lordship had been invited to take the chair because of his being lord of the manor of Battersea, and he alluded in his opening remarks to his connection with the parish as being now a nominal one. Still he had a sincere interest in anything affecting the parishioners of Battersea, and he did not think he could show that interest better than by taking part in such a movement as that. What they had to do was to put on a permanent and independent footing the hospital which, owing to the wisdom and liberality of Canon Erskine Clarke, had been established among them for fifteen years. Some years ago he (the speaker) was on a committee of the House of Lords on the subject of London hospitals. They went through the whole subject, and they had before them evidence of the vast good which was done by hospitals in the metropolis—hospitals which not only afforded relief to suffering humanity, but also provided the finest school for medical and surgical knowledge in the world. (Hear, hear.) He recollected full well that one of the matters that seemed to deserve attention, and which required some consideration, was the location of hospitals and distribution of benefit of hospitals in the metropolis. Hospitals were concentrated on one side of the Thames. There were not many on the south side, and one of the recommendations of the committee was that if possible some efforts should be made to establish on the south additional hospitals. Now he imagined that although that was not the locality which had been most pointed out as needing hospitals it did need a hospital very much. (Hear, hear.) It had an enormous population; he was told something like 160,000 souls. The population had increased fourfold within thirty years. With a growing population of that kind he could imagine nothing more beneficial and more needed than the establishment of a good hospital. They had heard of cases of rich men, with luxurious houses, admirably-ventilated bed-rooms, and the best sanitary appliances, preferring to go to a hospital and be treated there. If they found such benefit from the skilled and trained nurses, from the eminent men who belonged to the medical or surgical school of a hospital, how much more must be found by those who were compelled by necessity to live in smaller houses—houses which were not suited for cases of long illness, and where appliances for recovery and treatment were not available? (Hear, hear.) Referring to the special needs of those living in that district, his Lordship mentioned that they were five or six miles from any of the large hospitals of London, but he would not dwell further on the subject, for it would be treated by those who had intimate local knowledge and knew the needs of Battersea and what had already been done there. He would leave the advocacy of the movement to them, and, if they had not a great meeting he hoped all who were present would be thoroughly in earnest in carrying out the good work. (Applause.)

Mr. J. BURNS, M.P., in the course of a long and able speech, submitted the following resolution:—

That since Bolingbroke Hospital has received accident cases it has become conclusively evident that a local accident hospital is urgently needed, and should be permanently established in the neighbourhood.

The resolution, said Mr. Burns, stated a fact. It briefly gave the result of past experience in that district, and affirmed both in the light of fact and experience that a hospital in that district was eminently desirable. A resolution putting it in that way, however, left him the opportunity of converting other people who were not satisfied with the brief terms of the resolution. After recognising the difference between demanding an institution and supplying it, Mr. Burns addressed himself to a point upon which a good deal turned during the progress of the meeting. The movement was confronted, he said, by two currents of public opinion. One demanded that the hospital should be free in every respect; the other (which was worth recognising, because it had at the present time a hospital on its side) was for a paid hospital with accident wards attached. Now it seemed to him that for the moment, and in the direction of establishing what the needs of the district demanded, beggars must not be choosers, and even if a public meeting might object either to a free or to a voluntary hospital, what they had to consider was the man who had been knocked down in the street, and who might say: "A plague on both your houses, I want my broken arm or fractured rib mended." (Applause.) Bolingbroke House did not start as a free institution, but as a paid institution, plus contributions. Last year, however, they had nearly a thousand cases gratuitously administered to in the hospital, and whatever might be their view as to a free hospital, on behalf of those thousand people he thanked the Bolingbroke Committee for having done their best to attend to them. (Hear, hear.) He had taken the trouble in his own way, and he thought with perhaps his usual amount of confidence, to make certain inquiries to strengthen the case proved by the experience of the Bolingbroke Hospital as to the enormous number of accidents the hospital did not treat, and which had to go to other hospitals. Taking up the coroner's report he found that he dealt with 224 cases last year. Many of those were cases that a hospital in every street would not have been able to deal with, but there were a few where immediate attention in a hospital might have saved the amputation of limbs or prevented much pain; and in this connection he threw out the suggestion that an ambulance with india-rubber tyres at every fire-station, with a smart horse, might well be provided by the County Council. (Hear, hear.) Going on to refer to the number of Battersea patients treated at the central hospitals, Mr. Burns said the figures he had gathered were to him, as a Battersea man, most appalling. At St. Thomas's last year there were 387 in-patients from Battersea and 2,868 out-patients, or a total of 3,255 cases. Of Westminster, St. George's, and fifteen other hospitals he had made inquiries, and he had come to the conclusion that he might safely allow to Battersea one-thirtieth of the number of accidents occurring in London every year, viz., 10,000. If, therefore, Bolingbroke House had ten times its present accommodation they could send to it between seven and ten thousand accidents, casualties, and minor things, now taken by the police and other people either to the infirmary or some public hospital. After citing other figures and referring to the large number of accidents occurring in the district on the railway systems, Mr. Burns addressed himself to the question, Where should the hospital be? He had found considerable disagreement on the point; but he pointed out that in Bolingbroke House they had an existing institution, and that it was admirably situated both as regards centrality and because of its being near Wandsworth Common, where the rattle of traffic was at a minimum. (Applause.) Then they had to face the question, which was the most critical of all—Where was the money to come from? He was not a millionaire. (Laughter.) He did not see millionaires, either embryonic or dormant, in Culvert Road or Latchmere Road. There was not an excessive or burning desire on the part of the very rich to come forward to assist either existing institutions or new hospitals. That was demonstrated by the fact that to-morrow there would be collections for the London hospitals. Probably that collection would not exceed (he hoped it might) £40,000, and when that sum had been collected there would be a deficit staring existing institutions in the face of £150,000 for the forthcoming year. That fact ought to seriously influence the people who wanted a rate-supported free institution built to-morrow morning in the centre of Battersea Park. (Laughter and applause.) Local authorities had not yet devised a means by which a grant in aid could be given for the support of public hospitals and institutions like the Bolingbroke Hospital, but he thought the day was not far distant when public opinion would insist upon the local authorities being given that power. (Hear, hear.) If they did not like State-aided institutions there was one way to prevent them. Let them pay over £600 to Canon Erskine Clarke, and they would throw over, for twelve months at least, any idea of appealing to the rates. (Laughter.) He believed the outcome of that meeting would be that men of all shades of opinion would come together and form a large representative committee who would thrash out the means whereby Bolingbroke Hospital might once more be put on its legs. (Applause.)

Mr. Duncan Milligan, F.R.A.S., seconded the resolution, and pointed out that they were worse off than either the chairman had stated or Mr. Burns had intimated. Bolingbroke Hospital received accidents from every part of the union, which had a population of 327,000 inhabitants. Mr. Milligan argued at some length against the taking of accident cases to the workhouse infirmary.

Mr. Matthews, who spoke from the body of the room, proposed such an alteration in the resolution as would pledge the meeting to the establishment of a free hospital. Mr. Burns had told them there were two currents of opinion in regard to hospitals, but he had not made it very clear as to which he was with. He (the speaker) was absolutely with the free. (Applause.) Hospital reform was needed now in this country if ever it was, and the time was ripe if they were sincere in the matter. The next day was Hospital Sunday, and they would have cant from all the pulpits, or the majority of them, on a matter which affected everyone. Hospital Sunday was, in his opinion, a disgrace to them. ("No, no.") He said it was a disgrace to them as Christian men and women—those of them who were Christian men and women. (Laughter.) If Jesus Christ was to come to London on the morrow and it was Hospital Sunday, He would say exactly as He did to the Scribes and Pharisees; He would say to the middle and upper classes, "Woe unto you, middle and upper classes; hypocrites." The speaker's remarks were greeted with laughter and hisses, which led him to say that the matter was not one for joking. They might depend upon it there would be a day of judgment. As far as he could gather, the opinion of the working classes was that they had had quite enough of the voluntary system. He proceeded to quote the Duke of Devonshire in favour of the State making hospital provision, and concluded by stating that the principal reason why he spoke was because 383,000 families were compelled to live in one-roomed houses in London. If that were true, no man with an open mind could go away from that meeting and believe in the voluntary system for hospitals.

Mrs. Gray (who also spoke from the body of the room) seconded the amendment. She argued in favour of the hospital being made general as well as free.

Mr. Maskery, representative of the temperance, trades, and friendly societies, having spoken in favour of the resolution, the amendment was put to the meeting, and a very large number of hands were held up in favour of it. A count being insisted upon, the Chairman stated the number at 114, while 63 voted against the amendment, a result which was greeted with loud cheers from the Socialistic supporters of Mr. Matthews and Mrs. Gray. The promoters of the meeting being thus placed in a difficulty, the Hon. Conrad Dillon suggested as a means out of the dilemma that words should be added to the amended resolution to the effect that until a free hospital could be established efforts should be made to develop, continue, and support Bolingbroke Hospital. This was seconded from the body of the room, the seconder expressing the opinion that the Member for Battersea should have agitated at the County Council in favour of the body getting power to acquire and maintain hospitals. He was remarking that Mr. Burns might have done more for his constituency when the Chairman called him to order for wandering from the subject. The resolution, as amended and enlarged, was then put to the meeting, and carried unanimously. The next resolution was in favour of the permanent establishment of Bolingbroke Hospital; of the committee of the hospital making the necessary arrangements; and of the governing body being composed of subscribers of one guinea and upwards. The latter clause elicited some opposition, and eventually it was omitted. A formal resolution urging upon the Board of Trade the importance of the hospital being established as a corporation under their regulations was adopted without dissent, and a somewhat lively meeting closed with a vote of thanks to the Chairman, proposed by Canon Erskine Clarke.

### CITY OF LONDON TRUSS SOCIETY.

At the annual festival dinner of this society held last week the Bishop of Derry, who presided, strongly urged its claims to public support. It was founded in 1807 by the eminent surgeon John Taunton, whose name should be perpetuated in letters of gold as a benefactor to the human race. Altogether more than half a million of persons of both sexes and all ages had benefited by its help. He asked his hearers to remember that the society, though it supplemented the work of hospitals, yet was not classed with them, and so did not share in the proceeds of Hospital Sunday. The subscription list amounted to about £600.

### WEST LONDON HOSPITAL, HAMMERSMITH.

The West London Hospital, Hammersmith, urgently needs extension, and it was to aid the funds for this purpose that a well-attended meeting was held at Devonshire House on June 12th, the Duke of Devonshire himself presiding; Princess Louise (Marchioness of Lorne), the Marquis of Lorne, the Duchess of Devonshire, Cardinal Vaughan, and many others were present. Mr. Balfour, it had been hoped, would have also been present, but he was unavoidably detained by Parliamentary duties.

The Chairman recapitulated the history of the hospital. Starting as a dispensary in 1856, in a small private house, its work and accommodation had gradually increased. A part of the present building was opened in 1871, and a further portion in 1883. The population of the district served by this institution had enormously increased, and the demand upon its beds was out of all proportion with its possible accommodation. The only remedy for this condition of things was a very considerable extension, and the committee were now anxious to raise enough money to double the number of beds available. He considered that only by a prompt response to such an appeal could the present voluntary system of supporting hospitals be justified.

The Marquis of Lorne proposed, and Cardinal Vaughan seconded, a resolution recommending the claims of the hospital for further support and sympathy, and another resolution was passed approving of the proposed extension.

Sir W. O. Priestly, in supporting this, spoke strongly in favour of the voluntary system in hospital work, deprecating any departure from it.

It was finally announced that the donations promised, exclusive of cash, amounted to £1,095 9s.; and votes of thanks were offered to Princess Louise for attending the meeting, and to the Duke of Devonshire for lending his house for the purpose.

### ROYAL MATERNITY CHARITY.

On June 13th the triennial dinner of this charity was held at the Albion Tavern, Dr. Robert Barnes presiding. The institution was founded 138 years ago for the provision of gratuitous medical aid and medicine to married women in their own homes. It has a staff of forty doctors and forty-two midwives, the latter being trained at the society's expense. During the past year 4,390 women were attended, and owing to a falling off in subscriptions a further debt of £900 was incurred, making a total of £1,700 owing to the bank. Dr. Barnes spoke warmly of the good work of the charity, and observed that in addition to the relief given to the poor it must not be forgotten that it also afforded an enormous scope for observation and increase of skill in this particular branch of medical work. The donations afterwards announced amounted to something over £800, including £50 from the Queen, and 50 guineas from the Corporation of the City of London.

### GROSVENOR HOSPITAL FOR WOMEN AND CHILDREN.

At the annual general meeting, held on June 12th, Sir Frederick Seager Hunt presided, and announced that he had the greatest pleasure in stating that the building account had been largely increased by a generous donation from Lady Kortright of £1,000, and that the same kind benefactor had this year given an additional sum of £7,000, on condition that building operations should be at once begun. The hospital was therefore about to start upon a new era of its existence, and a special appeal would be made to the public at large. It was not proposed to build a large or costly edifice, but a small hospital, replete with all that modern science could suggest in the way of comforts and safeguards. The report was unanimously adopted, and the usual votes of thanks closed the proceedings.

### HOSPITAL FOR EPILEPSY AND PARALYSIS.

The annual meeting of the governors and supporters of this hospital was held in the Portman Rooms, Dorset Street, on the afternoon of the 13th inst. Sir Horace Farquhar presided. The report of the committee of management stated that to the regret of the committee the receipts during the year had come short of the expenditure by £200. To meet this deficit it had been necessary to sell a portion of stock. In order to preclude the necessity of further diminishing the very small invested capital, £1,000 must be received during the current year. On annual subscriptions there had been a decrease of £30; there had been no legacies; donations showed the slight ncrease of £27. The number of in-patients during 1894 was 98, as against 114, 116, and 122 during the three preceding years. Only one death occurred during the year. The "attendances" in the out-patient department numbered 8,843, as against 9,014 in 1893; the out-patient department, however, had been closed for three weeks. The whole expenditure on maintenance was £90 more than in 1893, and on administration £24 less. In moving the adoption of the report, Sir Horace Farquhar expressed the earnest hope that it would not be necessary to sell out any more stock. Canon Duckworth seconded. An hospital such as that, which ministered to diseases which were the darkest and most mysterious, had, he said, a special claim upon the public. The Rev. H. G. Rosedale moved a vote of thanks to the committee of management, and Dr. Ogilvie seconded. On the motion of Captain Ellis, seconded by Dr. Althans, a vote of thanks was passed to Sir Horace Farquhar for presiding.

### THE "PASSMORE EDWARDS" COTTAGE HOSPITAL, WOOD GREEN.

The foundation-stone of the hospital formally opened at Wood Green on Saturday, 15th inst., was laid last August by Mr. Passmore Edwards. Having heard that a cottage hospital was desired, Mr. Passmore Edwards offered to give £2,000 for the purpose of erecting a suitable building if it could be shown that the inhabitants consisted chiefly of working men, and if the local subscriptions could be counted upon for the maintenance of a hospital. This liberal offer was received with enthusiasm, £150 having been (up to date of opening) received in subscriptions and donations towards the annual expenditure, which is estimated at £350. For the land fund £130 has been already raised with a view to buying the ground on which the hospital stands, for which £300 is the total required.

The hospital contains eight beds for patients, the wards being particularly bright and pretty and having polished floors. There is a small operation-room with painted walls, cemented floor, and skylights, and a cabinet or press of highly polished wood for instruments and dressings, specially designed for institutional use by Messrs. Maw, Son, and Thompson, of Aldersgate Street. Splints, cradles, and other appliances have a small room devoted to them near to the little dispensary. The matron's sitting-room and the office or waiting-room are situated one on each side of the entrance door, with the wards, nurses' room, and kitchens all on the ground floor, three bed-rooms and the box-room above. A small laundry stands at the back of the house, and there is a little detached building for a mortuary. The grounds behind and in front of the hospital admit of a good-sized flower and kitchen garden, and when these are laid out the environment will be as pretty as the building itself.

The furnishing has been done by the ladies of Wood Green, who raised funds by the aid of a bazaar, completely fitting it throughout. They are to be congratulated on the excellent taste which has made each small room perfect in its way, the matron's and patients' quarters being equally considered.

The matron, Miss Warren, is a fully-trained nurse of large experience, and a probationer and one servant form her resident staff. The funds at present permit only of a modest salary for the matron and board, without salary, for the probationer.

### Opening Ceremony.

Mr. Joseph Howard, M.P., presided at the function, which a large number of guests attended. The new building was gaily decorated with flags, and on the rough ground in front (the garden of the future) were massed the visitors. A limited number of chairs was provided, but many unseated spectators waited through the afternoon with the cheerful patience so pleasantly characteristic of crowds. The ceremony was fixed for half-past three, but it was quite an hour later when loud cheers announced the arrival of Mr. and Mrs. Passmore Edwards, who, it afterwards transpired, had been carried inadvertently to a wrong station.

After courteous apologies for unintentional delay had been made by the president, Mr. Lewis, the hon. secretary was called upon to read his report, but this he was unable to do; the only copy (which had already done service with various representatives of the press) was discovered to be for the moment mislaid. The statement was therefore perforce taken as read, the visitors being asked to look up the details in the papers.

The architect, Mr. Chas. Bell, was congratulated on the success with which he had carried out the plans, and he expressed his satisfaction at having the honour of presenting a silver key. With this Mrs. Passmore Edwards formally opened the front door, saying: "Ladies and gentlemen, I have great pleasure in announcing the hospital opened."

The Rev. J. Thomas then read prayers, after which Colonel Durrant proposed a vote of thanks to Mr. and Mrs. Passmore Edwards, remarking that although sundry suggestions had been made relative to the person by whom the ceremony should be performed, it was unanimously agreed that Mrs. Passmore Edwards was the lady above all others who could most fittingly be chosen to formally open Mr. Passmore Edwards' princely gift. In reference to the collection made a week before by the ladies, Colonel Durrant spoke with appreciation of the large proportion of the pence given by the very class of persons for whom the hospital was primarily intended.

Mr. Palmer, in seconding the vote of thanks, remarked that now the hospital had been given to them it lay with those present to promise never to let it be closed.

Mr. Ridgway, the treasurer, in calling attention to the languishing condition of London hospitals at present, and to the large deficit recently announced, expressed an earnest hope that there would never be a proportionate deficit at Wood Green. He thought that in a few years they should show Mr. Passmore Edwards that the small beginning now made with eight beds might lead to great developments in such a neighbourhood.

Mr. Passmore Edwards, in returning thanks for his wife, commented on her unwillingness to speak in public, though he testified to her having "a silver tongue" and remarkable eloquence at home. In explaining their delayed arrival, Mr. Edwards said they had gone through a murky hour of purgatory, from which they only emerged on hearing the kindly greeting of cheers when they reached the hospital, in place of the hisses which he had almost anticipated. He said that a stray letter of Colonel Durrant's had been the original means of drawing his attention to the need of a hospital at Wood Green.

Mr. Bradshaw proposed a vote of thanks to Mr. Howard for presiding, and agreed with him that it would be a lasting disgrace if they allowed the hospital to fail for want of due support.

Mr. Lewis seconded the vote of thanks, and then the visitors were admitted to the hospital, which was soon crowded in every department by eager and interested spectators.

### "HOSPITAL SUNDAY" MEETING AT WALWORTH.

In connection with the Hospital Sunday Fund movement Mr. Henry C. Burdett delivered an address on the 13th inst. in the parish room of St. Margaret's, Chatham Street, Walworth, on "Living Pictures from the Hospitals: what they do and what they are." The Rev. W. J. Phillips, vicar, presided, and there was a crowded attendance. Mr. Burdett said that out of a population of four millions 1¾ millions found their way into the hospitals and received medical relief either as in or out patients in the course of a year. Roughly speaking, it might be said that half the population of London depended upon the hospitals for medical relief. He disagreed with those who were of opinion that it was impossible to maintain the hospitals by voluntary means, and that they ought to come upon the rates. Their experience of such rate-aided hospitals showed that they suffered from relatively inefficient administration and control of expenditure, and lacked medical skill and nursing. As compared even with great Poor Law infirmaries, such as Notting-hill, Paddington, and Marylebone, which were admirable in every respect, the hospitals showed greater rapidity of recovery and a greater degree of comfort. He could not believe that they were going to show such little regard for the poor as to cease to act the Samaritan and leave to the State all that was sweetest and noblest in charity. The people were not doing their duty. Out of every sovereign needed the public only contributed 7s. 6d., the patients 1s. 6d., while the "dead hand," in the shape of legacies and interest, provided 10s. 6d. The other 6d. represented the deficit. If an extra £25,000 were contributed on Hospital Sunday this deficit would disappear. The address was illustrated by a large number of photographs thrown on the screen by a limelight lantern, depicting scenes in the hospitals.—*The Times*.

